# Recommendations Based on Evidence by the Andalusian Group for Nutrition Reflection and Investigation (GARIN) for the Pre- and Postoperative Management of Patients Undergoing Obesity Surgery

**DOI:** 10.3390/nu12072002

**Published:** 2020-07-06

**Authors:** Antonio J. Martínez-Ortega, Gabriel Olveira, José L. Pereira-Cunill, Carmen Arraiza-Irigoyen, José M. García-Almeida, José A. Irles Rocamora, María J. Molina-Puerta, Juan B. Molina Soria, Juana M. Rabat-Restrepo, María I. Rebollo-Pérez, María P. Serrano-Aguayo, Carmen Tenorio-Jiménez, Francisco J. Vílches-López, Pedro P. García-Luna

**Affiliations:** 1 Unidad de Gestión Clínica de Endocrinología y Nutrición, Hospital Universitario Virgen del Rocío, 41013 Sevilla, Spain; dr.antmarort@gmail.com (A.J.M.-O.); jpereira@cica.es (J.L.P.-C.); piagua@gmail.com (M.P.S.-A.); garcialunapp@yahoo.es (P.P.G.-L.); 2 Endocrine Diseases Research Group, Institute of Biomedicine of Seville (IBiS), 41007 Sevilla, Spain; 3 Unidad de Gestión Clínica de Endocrinología y Nutrición, Hospital Regional Universitario de Málaga/Universidad de Málaga, 29010 Málaga, Spain; 4 Instituto de Investigación Biomédica de Málaga (IBIMA), 29010 Málaga, Spain; jgarciaalmeida@gmail.com; 5 CIBERDEM (CB07/08/0019), Instituto de Salud Carlos III, 28029 Madrid, Spain; 6 Servicio de Endocrinología y Nutrición, Complejo Hospitalario de Jaén, 23007 Jaén, Spain; carmenarraizairigoyen@gmail.com; 7 Unidad de gestión Clínica de Endocrinología y Nutrición, Hospital Universitario Virgen de la Victoria, 29010 Málaga, Spain; 8 UGC Endocrinología y Nutrición, Hospital Universitario Valme, 41014 Sevilla, Spain; irles@us.es; 9 UGC Endocrinología y Nutrición, Hospital Universitario Reina Sofía, 14004 Córdoba, Spain; cmmerinomjmolina@hotmail.com; 10 Instituto Maimónides de Investigación Biomédica de Córdoba (IMIBIC), 14004 Córdoba, Spain; 11 U. Nutrición y Dietética. Hospital General, Linares, 23700 Jaén, Spain; bautimolina@hotmail.com; 12 Endocrinología y Nutrición, Hospital Universitario Virgen Macarena, 41009 Sevilla, Spain; juanamariarabat@gmail.com; 13 Servicio de Endocrinología y Nutrición, Hospital Juan Ramón Jiménez, 21005 Huelva, Spain; misabel.rebollo@gmail.com; 14 Endocrinology and Nutrition Clinical Management Unit, University Hospital Virgen de las Nieves, 18014 Granada, Spain; carmentenoriojimenez@hotmail.com; 15 Servicio de Endocrinología y Nutrición, Hospital Puerta del Mar, 11009 Cádiz, Spain; franvilchez1977@gmail.com; 16 GARIN Group Coordinator, 41007 Seville, Spain

**Keywords:** Bariatric surgery, obesity, nutrient deficiency

## Abstract

In order to develop evidence-based recommendations and expert consensus for nutrition management of patients undergoing bariatric surgery and postoperative follow-up, we conducted a systematic literature search using PRISMA methodology plus critical appraisal following the SIGN and AGREE-II procedures. The results were discussed among all members of the GARIN group, and all members answered a Likert scale questionnaire to assess the degree of support for every recommendation. Patients undergoing bariatric surgery should be screened preoperatively for some micronutrient deficiencies and treated accordingly. A VLCD (Very Low-Calorie Diet) should be used for 4–8 weeks prior to surgery. Postoperatively, a liquid diet should be maintained for a month, followed by a semi-solid diet also for one month. Protein requirements (1–1.5 g/kg) should be estimated using adjusted weight. Systematic use of specific multivitamin supplements is encouraged. Calcium citrate and vitamin D supplements should be used at higher doses than are currently recommended. The use of proton-pump inhibitors should be individualised, and vitamin B12 and iron should be supplemented in case of deficit. All patients, especially pregnant women, teenagers, and elderly patients require a multidisciplinary approach and specialised follow-up. These recommendations and suggestions regarding nutrition management when undergoing bariatric surgery and postoperative follow-up have direct clinical applicability.

## 1. Introduction

Obesity is a pathology that has reached epidemic proportions in recent years. Apart from treatment using hygienic-dietary measures and drug therapy, surgery is also highly effective in the case of grade 3 obesity (BMI > 40 Kg/m^2^), or grade 2 obesity (BMI > 35 Kg/m^2^) with associated comorbidities. The main techniques currently in use are divided into: restrictive practices (those that restrict gastric net volume, known as the gastric sleeve, SG, sleeve gastrectomy or Laparoscopic Sleeve Gastrectomy, LSG); malabsorptive practices (those that achieve a malabsorption of nutrients, such as Biliopancreatic Diversion); and mixed practices (those that combine both procedures, as in the case of the gastric bypass, RYGBP) [1,2]. Currently, the almost universal access route is Laparoscopic. These techniques are highly effective in terms of weight loss; however, there are aspects that still do not have clear answers, such as indications in special groups, pre- and postoperative nutritional management, supplementation needs and the necessary tests in the follow-up of these patients. The Andalusian Group for Nutrition Reflection and Investigation (GARIN) aims to answer these questions, in an attempt to try to improve care for these patients and to standardize routine clinical practice.

## 2. Materials and Methods

The GARIN group members held a virtual meeting to propose and select questions related to the clinical practice and management of obese patients undergoing obesity surgery. In their opinion, these questions were of interest either because clinical practice guidelines (CPGs) did not provide a response or the response provided was unclear. Once selected, a systematic bibliographic search was carried out in PUBMED, Web of Science (WOS) and SCOPUS, which was narrowed down to systematic review articles, meta-analyses, controlled Clinical Trials (CTs), case series and CPGs, published in the last 10 years, limited to human beings, in English and Spanish. The keywords searched for were “bariatric surgery”, “gastric sleeve”, “Roux-en-Y gastric bypass”, in combination with “elderly”, “very low calorie diet”, “calcium”, “iron”, “micronutrients”, “vitamins”, “deficiency”, “proton-pump inhibitors”, “type 2 diabetes”, and “metabolic syndrome”. Unsystematic reviews, studies with less than 5 participants and publications in other languages were excluded.

**Figure 1:**
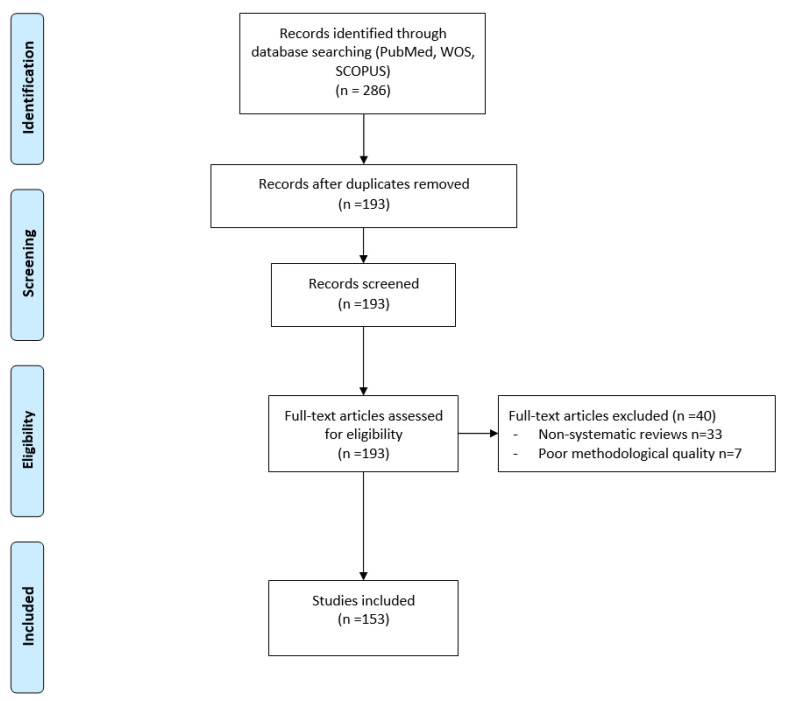
Flow diagram following the PRISMA methodology that reflects the selection and evaluation process of the analysed papers.

A total of 286 results were obtained, of which 193 results met the search criteria, after eliminating duplicates. Figure 1 specifies the process according to the PRISMA (Preferred Reporting Items for Systematic reviews and Meta-Analyses) methodology [3]. The critical evaluation of each article was performed using the SIGN methodology (Scottish Intercollegiate Guidelines Network), with the check list suited to each type of article and classified accordingly (Table 1 and Table 2) [4]. In the case of CPGs, the “AGREE” tool (Appraisal of Guidelines Research and Evaluation) was used in its second validated version in Spanish (AGREE-II). The articles were reviewed by two independent reviewers (AJMO and JLPC). In the case of doubt or discrepancy between reviewers, a third reviewer (PPGL) was used. 

The results were presented and discussed in two face-to-face meetings held with the members of the GARIN group. A consensus was reached regarding the responses to the previously selected questions, taking into consideration the evidence available. After the discussion, the group members electronically evaluated the consensus using a Likert-type scale [5,6]. The evaluation form had five possible answers to evaluate each recommendation (“totally disagree” with an assigned value of 1, “disagree” with an assigned value of 2, “neither agree nor disagree” with an assigned value of 3, “agree” with an assigned value of 4, and “totally agree” with an assigned value of 5). The consensus level of each recommendation was calculated by adding the total value resulting from the responses obtained, dividing it by the maximum value, and then multiplying it by 100. Finally, the draft of the article was circulated among all the members until the final version was achieved. 

## 3. Results

The article includes the responses obtained from the above-described process to twenty questions framed in three groups, according to the clinical situation of the bariatric patient, in the Preoperative period, during the Postoperative period or in the Obesity Surgery period in special situations. A list of the study articles used to respond to these questions, and the results of the critical evaluation of each article, is shown in Table 3.

All the GARIN group members responded to the survey. No “disagree” or “strongly disagree” responses were obtained. The questions and the results obtained are specified below.

## 4. Discussion 

### 4.1. Preoperative Management

#### 4.1.1. Should Micronutrient and Vitamin Levels Be Measured Preoperatively in Patients Who Are Candidates for Obesity Surgery?

The GARIN group is a group that seeks improvement to obese patient management. One area of concern is the ‘preoperative’ evaluation of potential candidates for surgery. Many of the reviewed guidelines concentrate on the ‘postoperative’ management of micronutrient deficits, but there is a lack of evidence for the preoperative setup. Vitamin and micronutrient deficiencies are often present prior to surgery, as several studies have reported [34,62,147,153,161]; however, the routine preoperative evaluation of these elements is a rarely seen requirement in local clinical protocols. Therefore, should these deficits be routinely screened for and treated if present? The recently published AACE/TOS/ASMBS/OMA/ASA 2019 guideline [161] (a high-quality guideline which was not included in the reviewed papers as it was published in April 2020, after the bibliography search was concluded) supports preoperative screening of vitamin B12, D and folate. However, what about chromium or other trace elements?

The GARIN group considers that it is convenient to determine the levels of certain micronutrients and vitamins preoperatively in patients who are candidates for Obesity Surgery. In some studies (Gregory DM et al., De Luís et al., Gomes de Lima et al., Wolf et al., Van Rutte et al.) it has been found that there is a deficiency of micronutrients and vitamins not only postoperatively but also preoperatively (Level of Evidence 3, grade of recommendation D) [34,62,147,153,162]. The GARIN group recommends that a vitamin D (25-Hydroxycholecalciferol) study and an iron metabolism study are performed at least once preoperatively on all patient-candidates for Obesity Surgery, plus vitamin B12 and folic acid studies in the risk population (elderly patients, those with a history of atrophic gastritis, patients with a low consumption of fruits and vegetables, or patients with DM treated with metformin, among others), and its repetition until surgery should be individualized as required by routine clinical practice (grade of recommendation D). This recommendation is in line with the proposal by American guidelines [93,161]. 

Consensus level: 93%.

#### 4.1.2. Should Deficits Be Supplemented? Are There Any Deficits that Have Been Shown to Be Critical?

If deficits are present, both clinical practice and the potential benefits indicate the reasonableness of treating them. The GARIN group experts advocate the use of supplements if any preoperative deficits exist (Level of Evidence 4, grade of recommendation D). 

Vitamin D deficiency is highly frequent in the obese population preoperatively (De Luís et al., Pereira-Santos et al., Van Rutte et al., etc.), so it must be assessed and treated (Level of Evidence 1++, grade of recommendation A) [34,113,147]. Regarding vitamin D supplementation, given the lack of solid evidence about the value to treat, the GARIN group experts advocate supplementation in all patients with values below 20 ng/mL, and in an individualized manner for values between 21 and 30 ng/mL. The pleiotropic effects of vitamin D at the level of carbohydrate metabolism and its possible role in the prevention of infectious processes, in addition to it reducing the risk of fractures and improving bone mineral density must be taken into account.

Iron, folate and ferritin deficiency are also frequent (Level of Evidence 3, grade of recommendation D). If deficits are detected the GARIN group experts recommend following the current treatment recommendations [163]. 

Consensus level: 100%

#### 4.1.3. Is the VLCD (Very Low-Calorie Diet) Recommended Preoperatively in All Patients? Is Liquid VLCD Better Than Solid VLCD?

The use of VLCDs (Very Low-Calorie Diets) in the preparatory preoperative phase to achieve rapid weight loss (and thereby reduce total liver volume) is still a cause of debate. Should liquid or solid VLCDs be used? And for how long? 

A VLCD, as its name indicates, is a diet with an important restriction in its caloric content (between 450 and 800 kcal per day). Its objective is to achieve weight loss in patients who due to their circumstances are unable to achieve satisfactory weight loss with standard restrictive diets, or in situations that require significant weight loss in a short period of time. This type of diet not only includes 0.8–1.5 g of proteins of high biological value per kilogram of ideal weight, but also the recommended daily doses of vitamins, minerals, trace elements and essential fatty acids, in order to obtain an adequate nutrition with conservation of lean mass. The VLCDs play a key role both pre and postoperatively in Obesity Surgery [15,79,146,164]. 

The GARIN group believes that a VLCD is useful preoperatively, as long as: it is managed by specialists with sufficient experience in its use; it provides a sufficient quantity of high biological value proteins; and that the subsequent control follows the specific recommendations in force (ECGs, monitoring of glucose metabolism and hypoglycaemic and hypotensive treatment, and regular analytical control, among others). Table 4 shows the main characteristics of a preoperative VLCD [165]. 

Both solid and liquid VLCDs allow similar weight losses, but patients who receive a preoperative liquid VLCD appear to have a greater loss of visceral fat mass, which is accompanied by a shorter surgical time, although this does not reflect on a reduction of complications (Leite-Faria et al., Level of Evidence 1−, grade of recommendation B) [79]. Although VLCD is not mentioned in European or American guidelines, the latter guidelines do highlight that a preoperative weight loss might decrease liver volume, and this in turn may help improve the technical aspects of surgery in patients with an enlarged liver or fatty liver disease [161]. 

The preoperative VLCD diet is not associated with greater postsurgical deficits (Benassar Remolar et al., Level of Evidence 3, grade of recommendation D) [15].

Given this evidence, the GARIN group experts advocate the use of a liquid VLCD diet preoperatively, for a recommended 4–8 weeks minimum prior to surgery and ideally for a longer length of time in patients with a higher BMI and/or associated metabolic-type comorbidities (such as HBP, DM, etc.), especially when taking into consideration its effects on better surgical results and greater visceral fat loss [79,166,167,168,169]. 

Consensus level: 91%.

References of the reviewed study articles in this section: [15,20,34,43,61,62,76,77,78,79,89,93,101,102,108,113,115,116,128,129,136,146,147,150,153].

### 4.2. Postoperative Management

When is it possible to resume solid food intake after Obesity Surgery?

The timing for resuming the intake of solid food after a successful surgical procedure is a matter of debate. The clinical opinion varies greatly, with time ranges that span from weeks to months; and as the aforementioned 2019 guidelines highlight, there is even a lack of consensus regarding the number of phases to be included [161].

The GARIN group advocates a liquid diet during the first postoperative phase, followed by a phase of purées, low in carbohydrates and rich in fibre. An adequate supply of liquids and at least 1–1.5 g of protein per kg of adjusted weight per day, together with micronutrients, vitamins and trace elements, must be ensured in both phases. There is controversy regarding the duration of each phase, with a lack of strong evidence in any direction. Although the rapid start of a solid diet in RYGBP (within the first postoperative week) was not associated with an increase in severe surgical complications in one of the studies considered (Edholm D, Level of Evidence 3) [38], which would reinforce the safety of the early start of the post-surgical diet, the degree of evidence is still insufficient to recommend a change in routine clinical practice. Thus, the GARIN group advocates maintaining a liquid diet for about 4 weeks, and then a semi-solid diet for another 4 weeks (grade of recommendation D). 

Consensus level: 84%.

#### 4.2.1. What Protein Provisions Should the Diet Contain?

Almost all the guidelines and systematic reviews analysed coincide in the need for a stable protein delivery, but differences arise in the finer details. The protein provision should ensure the prevention of muscle wasting and loss of lean mass. Protein intake is lower in the immediate postoperative period and at 6 and 12 months postoperative with respect to baseline values (and this trend seems to follow medium-term), therefore a higher dietary provision of protein should be recommended (Schiavo et al., Level of Evidence 3, grade of recommendation D) [130]. The GARIN group advises against calculating the protein provision based on a percentage of the diet’s total caloric value, since this method often results in insufficient intake. Instead, it is advisable to use a direct calculation based on the adjusted weight, at least 1 to 1.5 g of high biological value protein per kg of weight and day, as proposed by European and American guidelines (grade of recommendation D) [20,93,161]. Given that the necessary provisions are not normally reached with the usual intake, the use of protein supplements could be beneficial in the 6–12 months after surgery, allowing the maintenance of lean mass and functionality and improving fat loss (Schollenberger et al., Level of Evidence 1+, grade of recommendation B) [133].

Consensus level: 96%

#### 4.2.2. What Contributions of Calcium and Vitamin D Should Be Recommended Postoperatively?

The need for postoperative calcium and vitamin D supplementation is well established, as the evidence is robust. Bone metabolism and available vitamin D and calcium management often change after bariatric surgery, as bone turnover and bone density loss increases. Therefore, the use of these two compounds, either separately or in combination is a must. [31,65,81,104,120,154,156].

Deficits, especially of iron and vitamin D, as well as B12, are frequent in all techniques (the risk is even greater in the case of malabsorptive surgeries; Level of Evidence 1++). 

Elevated bone turnover, secondary hyperparathyroidism, and vitamin D deficiency, together with lower bone mineral density, have been observed in patients undergoing both LSG and RYGBP, with the deficit being greater in the latter group (Chakhtoura et al., Hsin et al., Liu et al., Level of Evidence 1++) [26,27,65,81]. Likewise, the risk of fracture is clearly increased (Zhang et al., Rousseau et al., Level of Evidence 1++) [122,160]. Thus, the use of calcium (1000 mg of calcium element at least) and vitamin D (880 IU of cholecalciferol) supplements is a standard postoperative recommendation (Flores et al., Liu et al., Level of Evidence 1++, grade of recommendation A) [44,45,81]. However, the current recommendations for calcium and vitamin D intake (1200–2000 mg/day of elemental calcium combined with 400–800 U of vitamin D, according to the European guideline [20]) seem to be insufficient for avoiding secondary hyperparathyroidism, so higher doses should be considered, especially after malabsorptive surgeries (Obinwanne et al., White et al., Wei et al., Level of Evidence 3, grade of recommendation D) [104,154,156]. In biliopancreatic diversion/Scopinaro surgeries the GARIN group recommends a higher intake of calcium (2000 mg/d) and especially a higher intake of vitamin D (2000 IU/d) (grade of recommendation D).

Consensus level: 91%.

#### 4.2.3. Should Iron Supplementation Be a Routine Postoperative Necessity?

Iron deficiency and anaemia in the postoperative situation is frequent and a cause for concern especially in younger female patients with polymenorrhagia or other medical conditions that alter the iron metabolism. This complication could, theoretically, be averted if we systematically supplement iron, but… is it worth it? Should we indiscriminately provide iron salts to every patient? Or should we perhaps adopt a more conservative approach, monitoring iron metabolism, with treatment being subject to having established the existence of iron deficiency? The 2017 European guidelines recommend prophylactic empiric iron supplementation after gastric bypass, biliopancreatic diversion, duodenal switch and sleeve gastrectomy [20]. However, providing specific iron supplementation in a systematic way does not seem to reduce the risk of anaemia (Kwon et al., Level of Evidence 1++, grade of recommendation grade A) [75]. However, since more than 40% of patients may have severe iron deficiencies, their levels should be monitored and treated appropriately, either by using oral iron or intravenous iron in the case of refractoriness (Obinwanne et al., Level of Evidence 3, grade of recommendation D) [105]. The GARIN group proposes periodic monitoring of iron levels and treatment in the case of deficit, coinciding with the American guidelines [93,161]. 

Consensus level: 85%.

#### 4.2.4. Should Vitamin B12 Supplementation Be a Routine Postoperative Necessity?

Insufficient vitamin B12 is a common occurrence in almost all clinical techniques. It can lead to neurological and hematological complications if left unchecked. The routine postoperative use of B12, to prevent the development of B12-induced anaemia, is supported by some authors and studies [75]. Several guidelines recommend the use of oral vitamin B12 supplements after gastric bypass, sleeve gastrectomy, biliopancreatic diversion and duodenal switch, with the possibility of parenteral administration of variable regimes [20,161].

The use of postoperative prophylactic vitamin B12 seems to prevent the development of anaemia (Kwon et al., Level of Evidence 1++, grade of recommendation A) [75]. The GARIN group only supports the parenteral treatment of vitamin B12 if the deficit is major (grade of recommendation D).

Consensus level: 85%.

#### 4.2.5. Should an Increase of the Dietary Intake of Other Micronutrients Be a Routine Postoperative Necessity?

The various surgical techniques intended for obesity treatment, and especially those that cause a degree of intestinal malabsorption, can lead to a deficit in trace elements, as previously stated [2,93]. This fact brings into play the need to improve the plasma profile of these elements. A dietary means to resolve this is a supplement of multivitamin pills and/or pharmacological preparations. We did not find any evidence regarding this matter, and there is no recommendation in any of the revised guidelines. Although there is no scientific evidence, a consideration of the pathophysiological mechanism of Obesity Surgery, especially malabsorptive surgery, would make an increase of the dietary intake of other micronutrients, including supplements recommendable (Level of Evidence 4, grade of recommendation D).

Consensus level: 89%.

#### 4.2.6. What Iron and Calcium Preparations Should Be Used?

The physiopathological changes in the digestive process can alter the absorption of several pharmacological compounds including calcium and iron salts and reduce their bioavailability. However, there are few differences between these formulations in terms of pharmacokinetic behaviour. This leads to the question of whether any form of iron or calcium salt is less influenced by these changes, meaning that it would be superior to the rest in achieving therapeutic goals? There is little evidence available regarding which iron preparation to use, and routine clinical practice is highly heterogeneous, so we cannot make a recommendation in this regard. Regarding calcium, the citrate preparations seem to have a higher bioavailability, especially in patients undergoing RYGBP (Sakhaee et al., evidence 1+, grade of recommendation B) [124,125]. The GARIN group recommends their use, as proposed in the European guidelines and in the American consensus (although therein the grade of recommendation is lower) [20,161].

Consensus level: 93%.

#### 4.2.7. What Analytical Determinations Should Be Made in the Follow-Up of Patients Who Have Undergone Obesity Surgery? How Often? 

Bariatric patient follow-up is currently based on expert opinions, and can vary to a high degree. Most guidelines draw a clear line between restrictive and malabsorptive surgeries with specific recommendations, but while some procedures and visit schedules might be similar in the short-term after surgery, in the long-term (6–12 months), the positions of the different groups start to diverge. For example, 24-hour urine calcium is recommended in the follow-up of RYGBP in the 2017 European Association for the Study of Obesity statement, while in the American 2019 guidelines it is not recommended [20,161]. Therefore, what is the best course of action? To do or not to do? Should we follow one lead or the other? What evidence do we have to support or reject any of them?

There is no evidence available to make a specific recommendation (expert consensus in most CPGs) about the frequency of determinations or what determinations to request. The various experts seem to agree that they differ depending on the technique used. 

Taking into account the postoperative deficits observed in the different studies (Alexandrou et al., Chakhtoura M et al., Kornerup, Kwon, Obinwanne) together with the consensus of experts, the GARIN group advocates the following analytical determinations, as shown in Table 5 [10,26,74,75,105]: 

Consensus level: 89%

#### 4.2.8. Is the Systematic Use of Proton Pump Inhibitors (PPIs) Necessary?

A frequent complication after bariatric surgery is the development of anastomotic ulcers. Its incidence varies according to different reports (0.6 to 25 %). Patients may present with perforation or massive bleeding after an asymptomatic onset. Other, less acute symptoms are epigastric burn and/or vomiting [28]. Proton pump inhibitors are one of the first steps in the treatment of these ulcers, but… should we use them systematically?

According to the latest American consensus, prophylactic therapy with proton-pump inhibitors should be considered for 90 days to 1 year, depending on risk; there is no specific recommendation in the 2017 European guidelines [20,161]. In RYGBP, the prophylactic use of PPIs significantly reduces the risk of ulcers, so it should be advised in all patients (Coblijn et al., Wu Chao et al., Level of Evidence 1++, grade of recommendation A) [28,157].

In LSG, we have found no evidence for or against its systematic use, so the GARIN group recommends individualising its use (Level of Evidence 4, grade of recommendation D).

We have not found any evidence regarding the duration of treatment with PPI for either technique, so we again recommend individualising its use (Level of Evidence 4, grade of recommendation D).

Consensus level: 96%.

#### 4.2.9. Is Systematic Supplementation with Multivitamin Complexes and Micronutrients Necessary?

According to the 2017 European guidelines and the newest American consensus, systematic supplementation with multivitamin complexes is recommended (grade of recommendation D and B, respectively) [20,161].

The GARIN group advocates the systematic use of multivitamin complexes. Taking into account the high prevalence of postsurgical deficits of micronutrients both in RYGBP and in LSG, even after 13 years of surgery (Ben-Porat et al., Gesquiere et al., Gillon et al., Gobato et al., Moizé et al., Obeid et al., Level of Evidence 3) [14,51,52,57,103] and that multivitamin complexes seem to reduce them (Gregory et al., Schijns et al., Level of Evidence 2+) [62,131], it seems reasonable to recommend the systematic use of multivitamin and mineral complexes (Level of Evidence 2+, grade of recommendation C).

Consensus level: 98%.

#### 4.2.10. Does the Composition of Currently Available Multivitamin Supplements Fit the Recommendations?

All guidelines support the use of multivitamin supplements in the bariatric patient, and in view of the aforementioned discussion, this seems reasonable. However, are all these supplements the same? Do they have similar composition and characteristics? And more importantly, do they comply with the current recommendations?

Although supplementation reduces micronutrient deficits, there are still patients who present deficits despite them (Ben-Porat et al., Dogan et al., Gesquiere et al., Guillon et al., Gobato et al., Moizé et al., Obeid et al., Level of Evidence 3) [14,36,51,52,57,98,103]. Therefore, we can assume that although in many cases it is sufficient, in others the supplements are insufficient, fundamentally because in many cases the composition does not adapt to the international recommendations (Table 6). Specific supplements appear to be more effective than non-specific supplements, so they should be recommended (Schijns et al., Level of Evidence 2+, grade of recommendation D) [131]. The GARIN group recommends, whenever available, those supplements that are specifically designed for patients undergoing Obesity Surgery.

Consensus level: 85%.

#### 4.2.11. Is It Necessary to Keep an Eye Out for the Appearance of Kidney Stones? What Measures Should Be Used to Prevent Their Appearance?

Another frequent and sometimes forgotten complication of bariatric surgery is the development of kidney stones, especially in RYGBP (11%); in LSG the incidence is significantly lower (1.5%). The underlying pathophysiological mechanisms of kidney stone formation following bariatric surgery are complex and diverse, including hyperoxaluria, hypocitraturia, and abnormally acid urine [170]. Currently, only 24-hour urinary calcium is recommended as a screening tool in most CPGs, with no specific indications for screening or routine detection of kidney stones in patients undergoing RYGBP. Therefore, we discussed how could we face this clinical problem.

At least in the RYGBP, a higher incidence of kidney stones and calcium supersaturation in urine has been found, so it is necessary to keep an eye out for its appearance (Upala et al., Level of Evidence 1+, grade of recommendation B) [145]. There is no evidence available for LSG.

There are no specific recommendations for screening or routine detection of kidney stones in patients undergoing RYGBP. It would be advisable to monitor the kidney function with the use of serum creatinine and formulas to estimate glomerular filtration, occasional 24-hourour urine calcium, and, in selected cases, imaging tests (Level of Evidence 4, grade of recommendation D) would be desirable.

Consensus level: 84%.

#### 4.2.12. Is Long-Term Follow-Up of Obesity Patients Who Have Undergone Restrictive Techniques Necessary? For How Long? Is Long-Term Follow-Up of Obesity Patients Who Have Undergone Malabsorptive Techniques Necessary? For How Long? Is Long-Term Follow-Up of Obesity Patients Who Have Undergone Mixed Techniques Necessary? For How Long?

Restrictive techniques have a significantly lower risk of deficiencies in comparison with malabsorptive techniques. Most guidelines advocate almost lifelong specialised follow-up of these patients, at least once a year. Nevertheless, in LSG patients who show no evident deficits, adequate clinical evolution and no severe comorbidities, this may not be necessary. These patients can be monitored by a general practitioner, thus reducing the burden on healthcare systems.

There is no high-quality evidence in this regard. After one year post-surgery, the GARIN group recommends annual check-ups in Specialised Care for at least five years of all patients who underwent Obesity Surgery. After this time, it is advisable to maintain annual check-ups in patients who underwent malabsorptive techniques, while those patients without complications who underwent purely restrictive techniques do not require specialised follow-up, except in selected cases (Level of Evidence 4, grade of recommendation D). 

Consensus level: 87%.

References of the studies reviewed in this section: [7,10,11,12,13,14,16,17,18,20,21,22,25,26,28,30,31,32,35,36,37,38,39,42,43,44,45,50,51,52,57,62,65,67,71,72,73,74,75,77,80,81,82,83,84,85,87,89,90,93,95,96,97,98,99,102,103,104,105,108,110,111,115,120,121,122,123,124,125,126,127,129,130,131,132,133,134,141,142,143,145,147,148,151,152,154,155,156,157,160].

### 4.3. Obesity Surgery in Special Situations

#### 4.3.1. What Technique Should We Recommend in Patients with Type 2 Diabetes Mellitus (T2DM) and Other Metabolic Comorbidities?

Type 2 Diabetes Mellitus (T2DM) is a great burden associated with obesity and perhaps one of the great pandemics of our time. One out of four morbidly obese patients have had T2DM, which is linked to higher cardiovascular risk and comorbidities. Bariatric surgery is a treatment option that can achieve diabetes remission, as several studies have demonstrated [159]. Both restrictive and malabsorptive techniques are effective in achieving this goal, but it is unclear if one is superior to the other.

Apparently, RYGBP and LSG are effective in terms of diabetes remission (especially in patients with a shorter duration of T2DM), but RYGBP seems to be superior in terms of probability of T2DM remission (RR 43.1 versus 16.6, Yska JP et al.), so it could be recommended as the first option (Level of Evidence 3, grade of recommendation D) [159]. The GARIN group recommends that, whatever the technique used, it should be performed by a Surgical Team with experience in that technique.

Consensus level: 91%.

#### 4.3.2. Pregnancy after Obesity Surgery

In women of childbearing potential, weight loss after bariatric surgery not only improves metabolic status, it also allows restoration of fertility, as this is often impaired in obese women. This phenomenon is thought to be associated with a steep reduction of insulin resistance and a decrease in androgen endogenous production [20]. However, bariatric surgery also causes different degrees of malabsorption and/or nutritional deficiencies, which could in turn cause foetal growth impairment, and in severe cases, miscarriage. As it is a complex situation, the intervention of several healthcare professionals such as endocrinologists, obstetricians, dietitians and/or specialised nurses, is required, to mention a few. Thus, proper preparation and close follow-up before and during pregnancy is of utmost importance for both mother and child [171]. 

Pregnancy after bariatric surgery is an increasingly frequent situation which does not have a large amount of scientific evidence available due to its peculiarities; therefore, the grade of recommendation is limited. However, it is a situation of risk for both the foetus (increased risk of small for gestational age (Adams et al., Level of Evidence 3) [9] and iron and vitamin A deficiency (Gimenes et al., Level of Evidence 3) [54]) and the mother (higher risk of anaemia (Gimenes et al., Level of Evidence 3) [53] and vitamin K deficiency (Jans G et al., Level of Evidence 3) [69]). Pregnant women who have undergone obesity surgery should be considered as high risk, not only due to nutrient deficiency but also due to complications of obesity prior to the surgery (Level of Evidence 4, grade of recommendation D). In experienced centres, however, the risk of complications is low and even comparable to the general population (González-Navarro et al., Jans G et al., Mead et al., Level of Evidence 3) [60,68,92].

All women who undergo Obesity Surgery when of childbearing age and who want to have a child should program their pregnancy in a regulated manner (Level of Evidence 4, grade of recommendation D), and receive specific supplementation with multivitamins and vitamin B12 in addition to folic acid and iodine (Jans G et al., Level of Evidence 2++, grade of recommendation C) [68]. There is no evidence regarding the minimum time to wait before programming a pregnancy after Obesity Surgery; the general opinion is at least one year after surgery and/or variations in body weight not exceeding 5% (Level of Evidence 4, grade of recommendation D). During this waiting time, effective contraceptive measures should be recommended (Level of Evidence 4, grade of recommendation D). 

All women, regardless of the type of surgery performed, should be screened for micronutrient deficiencies (Vitamin A, D, E, K, B12, folic acid and iron) at least every six-months prior to pregnancy, every three months during pregnancy (or sooner if any type of deficit is detected) and at 6-8 weeks post-partum, especially in the case of breastfeeding (Jans G et al., Level of Evidence 2++, grade of recommendation C) [68]. 

There is not enough evidence to make a recommendation in favour of or against the determination of zinc, iodine, magnesium, or calcium, but its monitoring seems reasonable (Level of Evidence 4, grade of recommendation D).

In addition to the standard supplementation it is advisable to include vitamin K supplementation, since low circulating levels of vitamin K have been observed in women who undergo Obesity Surgery (and even in pregnant women who do not undergo surgery) (Jans G et al., Level of Evidence 3, grade of recommendation D) [69]. 

There is no evidence found in the literature that contraindicates breastfeeding after delivery, so it can and should be recommended due to its benefits (Level of Evidence 4, grade of recommendation D).

In light of the above, the GARIN group recommends that:

(a) Pregnancy should be avoided in the first year after Obesity Surgery, and contraceptive measures should be routinely recommended.
(b) All women of childbearing age undergoing Obesity Surgery should program their gestation at an experienced centre.
(c) Possible micronutrient deficits (Vitamin A, D, E, K, B12, folic, iron) should be detected (AND treated) at least every six months prior to gestation, in each trimester of pregnancy (or sooner if any type of deficit is detected) and at 6-8 weeks post-partum, especially in case of breastfeeding.
(d) Breastfeeding is advocated.

Consensus level: 87%.

#### 4.3.3. Obesity Surgery in the Two Non-Working Age Groups: Adolescents and over 65 Years

The burden of obesity is not restricted to those aged between 18 and 65 years old. Sadly, we are witnessing a steep increase in childhood obesity, to an extent that we have never seen before nor could have predicted several years ago. According to the United States data, the rates of childhood obesity have tripled since the 1980s, and the prevalence of obesity in adolescents has quadrupled; 8.5% of youths meet the criteria of severely obese (BMI ≥ 120% of the 95th percentile), representing approximately 4.5 million children, who are at risk of developing T2DM, hypertension, dyslipidaemia or even sudden cardiac arrest in severe cases. This dramatic situation calls for immediate and vigorous measures to prevent devastating consequences for these children, and bariatric surgery, while not being the best solution, as it falls within the domain of tertiary prevention, is nevertheless a powerful weapon in our therapeutic arsenal [118].

Both LSG (Elhag et al., Level of Evidence 3) [41] and RYGBP (Souza-Silva et al., Olbers T et al., Level of Evidence 2++) [106,138] seems to be an effective technique for losing weight in adolescents between 13 and 18 years, and RYGBP seems to be effective in inducing remission of type 2 diabetes mellitus and resolving pre-diabetes, in the remission of hypertension and dyslipidaemia, and in the improvement of the quality of life (Souza-Silva et al., Olbers T et al., Level of Evidence 2++), meaning that it could be recommended in these patients (Level of Evidence 3, grade of recommendation D for LSG; Level of Evidence 2++, grade of recommendation C for RYGBP) [106,138]. 

There are no specific recommendations for the follow-up of this type of patient through to adulthood. It is advisable to maintain the same guidelines as for adults, in addition to monitoring the growth rate (Level of Evidence 4, grade of recommendation D). Special attention should be paid to the adolescent’s mental health, changes in their physique, and promotion of their integration (Level of Evidence 4, grade of recommendation D).

Obese patients older than 65 years of age are also a cause of concern. In Europe, in the year 2015, an estimated 32 million elderly people were reported to be obese, meaning that approximately 1.1 to 1.2% of the total elderly population suffered morbid obesity. These numbers, far from decreasing are currently on the rise. Although higher BMI value seems to predict a lower relative mortality in older adults (known as the “Obesity paradox”), up to the age of 75 the absolute mortality risk increases with increasing BMI. This could be explained by the presence of several confounding factors (survival effect, competing mortalities, shortened life expectancy, etc.) as highlighted in different studies, thus misleading to a false perception of obesity in the elderly [172,173]. 

Medical complications of obesity in this population group are mainly concentrated around metabolic syndrome (impaired glucose tolerance, hypertension, dyslipidaemia and cardiovascular disease). Bariatric surgery, as an effective tool for inducing remission of these conditions, is a very interesting option. However, the results in terms of weight loss seems to be less impressive than in younger patients, and the surgical risk is higher. As a result, the dilemma arises. Should we indicate bariatric surgery in elderly obese patients or not?

In those over 65 years, Obesity Surgery (both LSG and RYGBP) seems to have a greater number of complications, especially RYGBP, and to be associated with a lower rate of resolution of comorbidities (Giordano S, Haywood C, Marczuk P, Level of Evidence 1++) [55,63,88]. RYGBP also seems to be associated with higher mortality (Giordano S, Marczuk P, Level of Evidence 1++) [55,88]. Weight loss appears to be significantly less than in younger RYGBP patients (Giordano S, Marczuk P, Level of Evidence 1++) [55,88], although with LSG it seems that the loss is similar (Haywood C, Level of Evidence 1−) [63]. Consequently, RYGBP should be discouraged in people over 65, especially in cases of high cardiovascular risk (Giordano S, Marczuk P, Level of Evidence 1++, grade of recommendation A) [55,88], and LSG could be considered as an option (Haywood C, Level of Evidence 1−, grade of recommendation B) [63]. 

Consensus level: 87%.

#### 4.3.4. Obesity Surgery Re-operation. To Whom and with What Technique?

Although obesity surgery is highly effective, about 20% of bariatric patients fail to achieve a significant amount of weight loss (greater than 50% of their preoperative excessive weight), and/or experience medical or surgical complications (gastro-gastric fistula, recurrent ulcers), leading to a secondary surgical procedure [117].

There is no universal definition of surgery failure in terms of weight loss, so the various studies are quite heterogeneous, which in turn makes it difficult to generate high-level evidence (Mann P et al., degree of evidence 1+) [86]. The GARIN group must decide its own definition and extend its use to standardise criteria. 

However, it seems that the RYGBP after LSG is an effective and safe option, allowing additional weight loss and improvement of comorbidities (Tran D et al., Casillas RA et al., Ianelli A et al., Brethauer et al., Level of Evidence 1−, grade of recommendation B) [19,23,66,144], although weight loss is less when compared to patients who underwent RYGBP in the first instance (Pędziwiatr M et al., Level of Evidence 1++) [109]. With regard to RYGBP, the biliopancreatic diversion seems to be the most favourable technique, although evidence is scarce (Brethauer et al., Level of Evidence 1−, grade of recommendation B).

Consensus level: 75%.

References of the studies reviewed in this section: [8,9,19,20,23,24,29,33,40,41,46,47,48,49,53,54,55,56,60,63,64,66,68,69,72,86,88,89,91,92,93,94,100,102,106,107,108,109,112,114,117,118,119,135,137,139,140,144,149,158,159].

A brief summary of all recommendations can be found in Table 7.

## 5. Conclusions

The GARIN group, after reviewing the evidence available, recommend that patients undergoing bariatric surgery should be screened preoperatively for micronutrient deficiencies and treated accordingly. Additionally, a VLCD should be used for 4–8 weeks prior to surgery. Postoperatively, a liquid diet should be maintained for one month, followed by a semi-solid diet for one month. Protein requirements (1–1.5 g/kg) should be estimated using adjusted weight. Systematic use of specific multivitamin supplements is encouraged. Calcium citrate and vitamin D supplements should be used at higher doses than are currently recommended. The use of proton-pump inhibitors should be individualised, and vitamin B12 and iron should be supplemented in the case of deficiencies. All patients, especially pregnant women, teenagers, and elderly patients require a multidisciplinary approach and specialised follow-up. These recommendations and suggestions regarding nutritional management when undergoing bariatric surgery and postoperative follow-up have direct clinical applicability. Nevertheless, new studies are needed in order to increase the quality of evidence and provide concrete answers to questions that are still unclear. 

## Figures and Tables

**Table 1:** Level of Evidence (LoE) assigned to each article according to its quality.

LoE	Interpretation
1++	High quality meta-analyses, systematic reviews of CTs, or high quality CTs with a very low risk of bias
1+	Well conducted meta-analyses, systematic reviews of CTs, or well conducted CTs with a low risk of bias
1−	Meta-analyses, systematic reviews of CTs, or CTs with a high risk of bias
2++	High quality systematic reviews of case control or cohort studies. Case control or cohort studies with a low risk of bias and a high probability that the relationship is causal
2+	Well conducted case control or cohort studies with a low risk of bias and a moderate probability that the relationship is causal
2−	Case control or cohort studies with a high risk of bias and a significant risk that the relationship is not causal
3	Non-analytic studies, e.g., case reports and case series
4	Expert opinion

Abbreviations: CTs: Controlled Clinical Trials.

**Table 2:** Degree of recommendation applicable to each consensus response according to the supporting evidence.

Grade of Recommendation	Interpretation
A	At least one meta-analysis, systematic review, or CT rated as 1++, and directly applicable to the guidelines target population; or A body of scientific evidence consisting of studies rated as 1+ and demonstrating overall consistency of results.
B	A body of scientific evidence including studies rated as 2++, directly applicable to the guidelines target population, and demonstrating overall consistency of results; or Extrapolated scientific evidence from studies rated as 1++ or 1+
C	A body of scientific evidence including studies rated as 2+, directly applicable to the guidelines target population and demonstrating overall consistency of results; or Extrapolated scientific evidence from studies rated as 2++
D	Scientific evidence level 3 or 4; or Extrapolated scientific evidence from studies rated as 2+

**Table 3:** List of study articles/guidelines used to respond to the 20 selected questions.

Articles Used to Answer Each Question				
First Author	Type of Study	SIGN/AGREE II Score	LoE	Reference
Aaseth E	Case series	No checklist required	3	[7]
Abdemur A	Case series	No checklist required	3	[8]
Adams TD	Case-control study	High quality (++)	2++	[9]
Alexandrou A	Transversal study	No checklist required	3	[10]
Aron-Wisnewsky J	Case series	No checklist required	3	[11]
Bailly L	Case series	No checklist required	3	[12]
Basfi-Fer K	Case series	No checklist required	3	[13]
Ben-Porat T	Case series	No checklist required	3	[14]
Benassar Remolar MA	Case series	No checklist required	3	[15]
Botella-Carretero JI	Case series	No checklist required	3	[16]
Botella Romero F	Case series	No checklist required	3	[17]
Boyce SG	Case series	No checklist required	3	[18]
Brethauer SA	Systematic review	Low quality (−)	1−	[19]
Busetto L	Practice guideline	74.53% Good quality	NA	[20]
Cabral J	Systematic review	Low quality (−)	1−	[21]
Caron M	Case series	No checklist required	3	[22]
Casillas RA	Case series	No checklist required	3	[23]
Chagas C	Case series	No checklist required	3	[24]
Chakhtoura MT	Systematic review	Acceptable (+)	1+	[25]
Chakhtoura MT	Systematic review	High quality (++)	1++	[26]
Chakhtoura MT	Systematic review	High quality (++)	1++	[27]
Coblijn UK	Systematic review	Acceptable (+)	1+	[28]
Coblijn UK	Systematic review	Low quality (−)	1−	[29]
Cosendey Menegati G	Case-control study	Acceptable (+)	2+	[30]
Costa TL	Case-control study	Acceptable (+)	2+	[31]
Dagan SS	Cohort study	Acceptable (+)	2+	[32]
Daigle CR	Case series	No checklist required	3	[33]
De Luis DA	Case series	No checklist required	3	[34]
Del Villar Madrigal E	Case series	No checklist required	3	[35]
Dogan K	Case series	No checklist required	3	[36]
Dunstan M	Case series	No checklist required	3	[37]
Edholm D	Case series	No checklist required	3	[38]
Edholm D	Case series	No checklist required	3	[39]
Elbahrawy A	Case series	No checklist required	3	[40]
Elhag W	Case series	No checklist required	3	[41]
Fashandi AZ	Case series	No checklist required	3	[42]
Ferreira-Nicoletti C	Pre-post study	No checklist required	3	[43]
Flores L	NRCT	Acceptable (+)	1+	[44]
Flores L	Case series	No checklist required	3	[45]
Froylich D	Case series	No checklist required	3	[46]
Fulton C	Case series	No checklist required	3	[47]
Gadgil MD	Case-control study	High quality (++)	2++	[48]
Gebhart A	Case series	No checklist required	3	[49]
Gesquiere I	Case series	No checklist required	3	[50]
Gesquiere I	Case series	No checklist required	3	[51]
Gillon S	Case series	No checklist required	3	[52]
Gimenes JC	Transversal study	No checklist required	3	[53]
Gimenes JC	Case series	No checklist required	3	[54]
Giordano S	Systematic review	High quality (++)	1++	[55]
Giordano S	Systematic review	Acceptable (+)	1+	[56]
Gobato RC	Case series	No checklist required	3	[57]
Goldberg HR	Case series	No checklist required	3	[58]
Gomes de Lima KV	Case series	No checklist required	3	[59]
González-Navarro I	Case series	No checklist required	3	[60]
Grace, C	Case series	No checklist required	3	[61]
Gregory DM	Pre-post study	No checklist required	3	[62]
Haywood C	Systematic review	Low quality (−)	1−	[63]
Homan J	Case series	No checklist required	3	[64]
Hsin MC	Cohort study	Acceptable (+)	2+	[65]
Iannelli A	Case series	No checklist required	3	[66]
James H	Case series	No checklist required	3	[67]
Jans G	Systematic review	Low quality (−)	2++	[68]
Jans G	Cohort study	Acceptable (+)	2+	[69]
Jáuregui-Lobera I	Systematic review	Acceptable (+)	1+	[70]
Kalani A	Systematic review	High quality (++)	1++	[71]
Kim MK	Case series	No checklist required	3	[72]
Kiyomi-Ito M	Systematic review	High quality (++)	1++	[73]
Kornerup LS	Case series	No checklist required	3	[74]
Kwon Y	Systematic review	High quality (++)	1++	[75]
Krzizek EC	Case series	No checklist required	3	[76]
Lecube A	Transversal study	No checklist required	3	[77]
Lefebvre P	Case series	No checklist required	3	[78]
Leite Faria, S	Open RCT	Acceptable (+)	1−	[79]
Li Z	Systematic review	High quality (++)	1++	[80]
Liu C	Systematic review	High quality (++)	1++	[81]
Lucas Soares F	Case series	No checklist required	3	[82]
Luger M	RCT	High quality (++)	1+	[83]
Majumder S	Systematic review	Low quality (−)	1−	[84]
Malone M	Case series	No checklist required	3	[85]
Mann JP	Systematic review	Low quality (−)	1−	[86]
Manousou S	Case-control study	High quality (++)	2++	[87]
Marczuk P	Systematic review	High quality (++)	1++	[88]
Martín García-Almenta E	Practice guideline	54.03% Low quality	NA	[89]
McCracken E	Case-control study	High quality (++)	2++	[90]
McGlone ES	Case series	No checklist required	3	[91]
Mead NC	Transversal study	No checklist required	3	[92]
Mechanick JL	Practice guideline	90.06% Excellent quality	NA	[93]
Mendes-Filho AM	Systematic review	Low quality (−)	1−	[94]
Mingrone G	Systematic review	Low quality (−)	1−	[95]
Mischler R	RCT	Low quality (−)	1−	[96]
Mischler R	Transversal study	No checklist required	3	[97]
Moizé V	Case series	No checklist required	3	[98]
Moore CE	Case series	No checklist required	3	[99]
Morales MP	Case series	No checklist required	3	[100]
Nicoletti CF	Case series	No checklist required	3	[101]
O’Kane M	Practice guideline	77.63% Good quality	NA	[102]
Obeid NR	Case series	No checklist required	3	[103]
Obinwanne K	Case series	No checklist required	3	[104]
Obinwanne K	Case series	No checklist required	3	[105]
Olbers T	Cohort study	High quality (++)	2+	[106]
Parmar C	Transversal study	No checklist required	3	[107]
Parrot J	Practice guideline	63.35% Acceptable quality	NA	[108]
Pędziwiatr M	Systematic review	High quality (++)	1++	[109]
Pellitero S	Case series	No checklist required	3	[110]
Pereira S	NRCT	Acceptable (+)	1−	[111]
Pereira Da Cruz S	Cohort study	High quality (++)	2+	[112]
Pereira-Santos M	Systematic review	High quality (++)	1++	[113]
Pérez Quirante F	Transversal study	No checklist required	3	[114]
Peterson LA	Systematic review	Low quality (−)	1−	[115]
Peterson LA	Transversal study	No checklist required	3	[116]
Pinto-Bastos A	Systematic review	Low quality (−)	1−	[117]
Pratt JSA	Practice guideline	70.8% Good quality	NA	[118]
Quezada N	Case series	No checklist required	3	[119]
Rodríguez-Carmona Y	Systematic review	High quality (++)	1++	[120]
Rottenstreich A	Case series	No checklist required	3	[121]
Rousseau C	Case-control study	High quality (++)	2++	[122]
Ruíz-Tovar J	Case series	No checklist required	3	[123]
Sakhaee K	RCT	Acceptable (+)	1+	[124]
Sakhaee K	RCT	Acceptable (+)	1+	[125]
Salgado W	Case series	No checklist required	3	[126]
Sallé A	Case series	No checklist required	3	[127]
Sánchez A	Transversal study	No checklist required	3	[128]
Santarpia, L	Case series	No checklist required	3	[129]
Schiavo L	Pre-post study	No checklist required	3	[130]
Schijns W	Cohort study	Acceptable (+)	2+	[131]
Schneider J	RCT	Acceptable (+)	1+	[132]
Schollenberger AE	RCT	Acceptable (+)	1+	[133]
Shah M	Case series	No checklist required	3	[134]
Sheng B	Systematic review	High quality (++)	1+	[135]
Sherf-Dagan S	Transversal study	No checklist required	3	[136]
Sjöström L	Cohort study	Acceptable (+)	2+	[137]
Souza Silva J	Pre-post study	No checklist required	3	[138]
Susmallian S	Case series	No checklist required	3	[139]
Susmallian S	Case series	No checklist required	3	[140]
Tang L	Case series	No checklist required	3	[141]
Tondapu P	RCT	Acceptable (+)	1+	[142]
Topart P	Case series	No checklist required	3	[143]
Tran DD	Systematic review	Low quality (−)	1−	[144]
Upala S	Systematic review	High quality (++)	1+	[145]
Van Nieuwenhove Y	RCT	Acceptable (+)	1+	[146]
Van Rutte PWJ	Pre-post study	No checklist required	3	[147]
Verger EO	Case series	No checklist required	3	[148]
Vinan-Vega M	Case-control study	Acceptable (+)	2+	[149]
Wang C	Case series	No checklist required	3	[150]
Wang FG	Systematic review	High quality (++)	1++	[151]
Ward EK	Retrospective cohort study	Acceptable (+)	2+	[152]
Wolf E	Transversal study	No checklist required	3	[153]
Wei JH	Pre-post study	No checklist required	3	[154]
Wang TC	Systematic review	High quality (++)	1++	[155]
White MG	Case series	No checklist required	3	[156]
Wu Chao Ying V	Systematic review	High quality (++)	1++	[157]
Yorke E	Case series	No checklist required	3	[158]
Yska JP	Case series	No checklist required	3	[159]
Zhang Q	Systematic review	High quality (++)	1++	[160]

Abbreviations: NA, Not Applicable; NRCT Non-Randomised Controlled Trial; RCT, Randomised Controlled Trial.

**Table 4:** VLCD main characteristics.

Total Caloric Value (Kcal/day)	450–800
Carbohydrate content	At least 55 gr/day
Protein content	50 gr/day (high biological value)
Lipids	7 gr
Linoleic acid	3 gr
Alpha-linolenic acid	0.5 gr
Fibre	10 gr
Vitamins, micronutrients and trace elements	100% of daily requirements

**Table 5:** Follow-up analytical recommendations.

	Pre-Surgery	1 Month	3 Months	6 Months	12 Months	Annual
CBC/Biochemistry	X	X	X	X	X	X
Albumin	X	X	X	X	X	X
Prealbumin	X	**Optional**	**Optional**	**Optional**	**Optional**	**Optional**
CRP	X	X	X	X	X	X
Iron/Ferritin	X		X	X	X	X
Ca/P/Mg	X		X	X	X	X
iPTH				X	X	X
B12/Folic acid	X	X *		X^A^	X^A^	X^A^
Vitamin D	X			X	X	X
Zn/Cu				**Optional**	**Optional**	**Optional**
B1				**Optional**	**Optional**	**Optional**
Vitamin A and E				X	X ^A^	X ^A^

* It is advisable to request in case of preoperative deficit. In other cases, optional. ^A^ Mandatory in malabsorptive surgery, optional in restrictive. X: must be done.

**Table 6:** Comparison between the composition of different multivitamins available in Spain.

	Recommendations of the American Society for Metabolic and Bariatric Surgery (ASMBS)	Multicentrum (Per tablet)	Multi-Tenex (Per tablet)	Supradyn (Per tablet)	Micebrina Complex
Vit A, μg	1500–3000	800	800	800	450
Vit B1, mg	1.2	1.4	1.4	1.1	10
Vit B12, μg	350–500	2.5	1	2.5	12
Vit D3, μg	75	5	5	5	10
Vit E, mg	15	15	10	12	30
Vit K, μg	90–120	30	-	25	-
Copper, mg	1–2	0.5	2	1	2
Iron, mg	45–60	5	14	14	18
Zinc, mg	8–22	5	14	10	15
Calcium, mg	1200–1500	162	100	120	0.15 (Calcium iodate)

Table prepared according to data provided by the manufacturer.

**Table 7:** Final recommendations/suggestions reached in the present review.

Recommendation	Grade of Recommendation/Consensus Level
It is convenient to determine the levels of certain micronutrients and vitamins preoperatively, at least vitamin D and iron metabolism. Folic acid and B12 vitamin should be included in certain populations	D/93%
Specific micronutrient supplements should be used if there is any evidence of any preoperative deficit following the current treatment recommendations	D/100%
We recommend the use of a liquid VLCD diet preoperatively, for at least 4-8 weeks minimum prior to surgery and ideally for a longer length of time in selected patients	B/91%
After surgery, a liquid diet should be maintained for about 4 weeks, and then a semi-solid diet for another 4 weeks	D/84%
The GARIN group advises against calculating the protein provision based on a percentage of the diet’s total caloric value, since this method often results in insufficient intake. Instead, it is advisable to use a direct calculation based on the adjusted weight, at least 1 to 1.5 gr of high biological value protein per Kg of weight and day	D/96%
The use of protein supplements could be beneficial in the 6-12 months after surgery	B/96%
The postoperative use of calcium (1000 mg of calcium element at least) and vitamin D (880 IU of cholecalciferol) supplements are recommended	A/91%
In biliopancreatic diversion/Scopinaro surgeries the GARIN group recommends a higher intake of calcium (2000 mg/d) and especially a higher intake of vitamin D (2000 IU/d)	D/91%
Periodic monitoring of iron levels after surgery should be performed, and in the case of deficit, treated accordingly	D/85%
Use of parenteral treatment for vitamin B12 deficiency only if the deficit is evident	D/85%
Although there is no scientific evidence, a consideration of the pathophysiological mechanism of Obesity Surgery, especially malabsorptive surgery, would make an increase of the dietary intake of other micronutrients, including supplements recommendable	D/89%
Calcium citrate preparations should be recommended above other calcium compounds, especially in RYGBP	B/93%
The GARIN group suggest periodic and customised analytical follow-up after surgery. Vitamin A, E, B12 and folic acid are mandatory in malabsorptive surgery (See text for details)	D/89%
The GARIN group recommends individualising the use and duration of PPI therapy	D/96%
We recommend the systematic use of multivitamin and mineral complexes	C/98%
The GARIN group recommends, whenever available, the use of supplements that are specifically designed for patients undergoing Obesity Surgery	D/85%
We recommend periodical kidney function monitoring using serum creatinine and specific formulas to estimate glomerular filtration, occasional 24-hourour urine calcium, and, in selected cases, imaging tests, at least in patients who underwent RYGBP to rule out kidney stones.	D/84%
After one year post-surgery, the GARIN group recommends annual check-ups in Specialised Care for at least five years of all patients who underwent Obesity Surgery. After this time, it is advisable to maintain annual check-ups in patients who underwent malabsorptive techniques, while those patients without complications who underwent purely restrictive techniques do not require specialised follow-up, except in selected cases	D/87%
The GARIN group recommends that in patients with T2DM, RYGBP should be considered before LSG; independently of the technique used, it should be performed by a Surgical Team with experience in that technique	D/91%
Pregnancy should be avoided in the first year after Obesity Surgery, and contraceptive measures should be routinely recommended	D/87%
All women of childbearing age undergoing Obesity Surgery should programme their gestation at an experienced centre	D/87%
Possible micronutrient deficits (Vitamin A, D, E, K, B12, folic, iron) should be detected (AND treated) at least every six months prior to gestation, in each trimester of pregnancy (or sooner if any type of deficit is detected ) and at 6-8 weeks post-partum, especially in case of breastfeeding.	C/87%
Breastfeeding is encouraged	D/87%
In selected adolescent population between 13 and 18 years LSG can be considered for weight loss	D/87%
In selected adolescent patients between 13 and 18 years, RYGBP can be considered for weight loss, especially in patients with prediabetes/T2DM, hypertension and/or dyslipidaemia	C/87%
BGYR should be discouraged in people over 65, especially in cases of high cardiovascular risk	A/87%
In selected patients over 65 years LSG could be considered as an option	B/87%
If re-intervention is needed due to poor results in weight loss, RYGBP after LSG is an effective and safe option, allowing additional weight loss and improvement of comorbidities. If the first surgery was RYGBP, biliopancreatic diversion seems to be the most favourable technique	B/75%
